# The spatial impact of a Western diet in enriching Galectin-1-regulated Rho, ECM, and SASP signaling in a novel MASH-HCC mouse model

**DOI:** 10.1186/s40364-024-00660-3

**Published:** 2024-10-14

**Authors:** Tahereh Setayesh, Ying Hu, Farzam Vaziri, Dongguang Wei, Yu-Jui Yvonne Wan

**Affiliations:** Department of Medical Pathology and Laboratory Medicine, University of California, Davis, Room 3400B, Research Building III, 4645 2nd Ave, Sacramento, CA 95817 USA

**Keywords:** Hepatocellular carcinoma, Metabolic-associated fatty liver disease, Epithelial-to-mesenchymal transition, Extracellular matrix remodeling, and senescence-associated secretory phenotypes

## Abstract

**Background:**

Hepatocellular carcinoma (HCC) arising from metabolic dysfunction-associated steatohepatitis (MASH) presents a significant clinical challenge, particularly given the prevalence of the Western diet (WD). The influence of diet on the tumor microenvironment remains poorly understood. Galectin-1 (Gal-1) is a biomarker for HCC and has a crucial role in liver carcinogenesis. Our previous studies demonstrated that silencing Gal-1 effectively treats mouse HCC. However, the impacts of a WD on Gal-1 signaling on MASH to HCC progression are unknown, and this study addresses these knowledge gaps.

**Methods:**

We developed a novel MASH-HCC mouse model. Using spatial transcriptomics and multiplex immunohistochemistry (IHC), we studied the effects of a WD on the liver and tumor microenvironment. By modulating Gal-1 expression through silencing and overexpression, we explored the location-specific impacts of WD on Gal-1 signaling.

**Results:**

Pathways such as Rho signaling, extracellular matrix (ECM) remodeling, and senescence-associated secretory phenotypes (SASP) were prominently activated in WD-induced metabolic dysfunction-associated fatty liver disease (MAFLD) and MASH-HCC, compared to healthy livers controls. Furthermore, Rho GTPase effectors, ECM remodeling, neutrophil degranulation, cellular stress, and cell cycle pathways were consistently enriched in human and mouse MASH-HCC. Spatially, these pathways were enriched in the tumor and tumor margins of mouse MASH-HCC. Additionally, there was a notable increase in CD11c and PD-L1-positive cells from non-tumor tissues to the tumor margin and inside the tumor of MASH-HCC, suggesting compromised immune surveillance due to WD intake. Moreover, MASH-HCC exhibited significant Gal-1 induction in N-Cadherin-positive cells, indicating enhanced epithelial-to-mesenchymal transition (EMT). Modulating Gal-1 expression in MASH-HCC further established its specific roles in regulating Rho signaling and SASP in the tumor margin and non-tumor tissues in MASH-HCC.

**Conclusion:**

WD intake significantly influences vital cellular processes involved in Gal-1-mediated signaling, including Rho signaling and ECM remodeling, in the tumor microenvironment, thereby contributing to the development of MASH-HCC.

**Supplementary Information:**

The online version contains supplementary material available at 10.1186/s40364-024-00660-3.

## Background

Metabolic dysfunction-associated steatohepatitis (MASH) is a major cause of hepatocellular carcinoma (HCC). Due to the consumption of the Western diet (WD), the prevalence of MASH continues rising, and HCC is the 3rd cause of cancer-associated death worldwide [1, 2]. There is no drug to prevent MASH, and the treatment options for HCC are very limited, and effective drugs are lacking. The goals of the current study are several: (1) establish and characterize a human-relevant MASH-HCC model; (2) understand how a WD affects the tumor environment, including the tumor itself, tumor margin, and non-tumorous tissue next to the tumor, and (3) targeting WD-linked signaling to understand MASH-HCC tumorigenesis and treatment.

A Western diet (WD) or a high-fat diet (HFD) that simulates individual preferences for carbohydrates or fat is often used to study metabolic liver diseases in animal models [3–5]. Compared to a WD, an HFD is much more efficient in promoting weight gain; in contrast, a WD is highly inflammatory [6]. Our studies reveal that short-term exposure to a WD for two weeks is sufficient to expand interleukin (IL)-17 A-producing γδ T cells. This triggers inflammation in both skin and joints, along with dysbiosis. Importantly, these effects can be reversed by diet-switching [7–9]. Long-term WD intake leads to MASH and impacts neuroplasticity [10]. The significance of IL17 signaling in liver carcinogenesis has been revealed. In HCC patients, increased Th17 cells are correlated with the HCC stage and tumor size [11, 12]. Our study revealed that overexpression of IL23/IL17 signaling exacerbates mouse liver carcinogenesis while inhibiting IL17 signaling contributes to positive HCC treatment outcomes [13]. Besides IL17, the IL-6/JAK2/STAT3 signaling plays a crucial role in the development and progression of HCC [14, 15]. It is important to understand further how a WD spatially affects the HCC tumor environment.

Complex interplays between inflammation and extracellular matrix (ECM) remodeling influence the tumor microenvironment [16]. In liver cancer, ECM remodeling is crucial in tumor surveillance, affecting growth [17, 18]. For example, matrix metalloproteinases create an immunosuppressive environment, and collagen facilitates the recruitment of immune cells to the tumor microenvironment [19, 20]. Moreover, a dense matrix physically hinders the infiltration and movement of T cells, impairing their ability to target cancer cells [21]. Thus, ECM remodeling has a pivotal role in liver carcinogenesis. Moreover, there are reciprocal relationships between ECM and epithelial-to-mesenchymal transition (EMT). ECM acts as a structural scaffold and a reservoir for EMT signaling. Specifically, increased collagen promotes EMT. Moreover, ECM components like transforming growth factor-βand matrix metalloproteinases can induce EMT [22, 23]. On the other hand, cells undergoing EMT change in their interaction with the ECM, allowing cells to break through the basement membrane and invade surrounding tissues, contributing to ECM remodeling and facilitating tumor cell migration [24, 25]. Whether a WD contributes to those tumorigenesis processes should be addressed.

Because of the inflammatory nature of a WD, we hypothesize that WD-associated MASH-HCC has heightened ECM and EMT signaling. Thus, a model of WD-MASH-HCC is expected to be useful in targeting both ECM and EMT. The current study has validated this hypothesis by establishing and characterizing a novel mouse MASH-HCC model. The molecular landscape of MASH-HCC vs. HCC, i.e., HCC arising in a healthy liver, has been compared spatially to identify the WD effects. Moreover, we have studied the spatial effects of silencing and overexpressing galectin-1 (Gal-1), which is pivotal in regulating EMT in this newly established MASH-HCC. Our data revealed the novel role of the WD in inducing Gal-1-regulated Rho, ECM, EMT, and senescence signaling.

## Methods

### Generating MASH-HCC and HCC

*FVB/N* male mice (Jackson Laboratories, Sacramento, CA, USA) were housed in standard filter-top cages at 22 °C under a 12-hour light on-and-off cycle. After weaning, the mice were fed a WD consisting of 21.2% fat, 34% sucrose, and 0.2% cholesterol (TD.140414; Harlan Teklad, Madison, WI) for five months. In one group, two months before euthanasia, the mice received a hydrodynamic injection of plasmids consisting of myr-Akt1 and N-RasV12 (1 µg/g body weight) and Sleeping beauty transposase (0.08 µg/g body weight) in 2 mL PBS [13, 26, 27]. Constructs utilized exhibited sustained gene expression via a hydrodynamic injection [28]. Mice received either a WD for five months to induce metabolic dysfunction-associated fatty liver disease (MAFLD) were used to compare with age- and gender-matched mice who received a healthy diet.

### Silencing and overexpressing Gal-1

To silence the expression of Gal-1, adeno-associated virus, serotype 9 (AAV9, Applied biological material, Richmond, BC, Canada) was used [26]. Gal-1 siRNA (5 × 10^10^ genome copy, one injection) was administered intravenously, and scramble-AAV9 was used as a control. The same vector was used for Gal-1 overexpression (5 × 10^10^ genome copy, one time) [26], and AAV9 without an insert was used as a control. Animal experiments were carried out following the National Institutes of Health Guidelines for the Care and Use of Laboratory Animals, with an approved protocol by the Institutional Animal Care and Use Committee of the University of California, Davis.

### Histology and multiplex-immunohistochemistry (IHC)

Tissues were fixed in 10% formalin. Hematoxylin and eosin (H&E) staining used paraffin-embedded liver Sect. (5-µm sections). Multiplex-IHC was conducted using fluorescence-based tyramide signal amplification using published methods [29]. The antibodies used were anti-E-cadherin (ECAD) (Cell Signaling Technology, Danvers, MA, USA), anti-N-cadherin (NCAD) (Epitomics, Burlingame CA, USA), anti-Gal-1 (Abcam, Fremont, CA, USA), and anti-F4/80 (Cell Signaling Technology, Danvers, MA, USA). ECAD, Gal-1, F4/80, and NCAD were detected using Opal fluorophores (Opal520, 620, 650, and 690: Akoya Biosciences) conjugated to tyramide, respectively. DAPI was used to stain nuclei. Fluorescence signals were scanned using the Vectra 3 Automated Quantitative Pathology Imaging System (Akoya Biosciences, Marlborough, MA, USA), and signal unmixing was executed utilizing the inForm software (Akoya Biosciences, Marlborough, MA, USA).

### RNA extraction, sequencing, and analysis

Hepatic RNA from healthy, HCC, and MASH-HCC samples was extracted using TRIzol reagent (Thermo Fisher Scientific, Waltham, MA, USA) for cDNA synthesis with a high-capacity kit (Applied Biosystems, Carlsbad, CA, USA). Following quality control using Qubit and Bioanalyzer instruments, libraries were prepared utilizing the NEBNext Ultra II non-directional RNA Library Prep kit [13, 26]. The resulting *p-*values were adjusted following Benjamini and Hochberg’s approach to control the false discovery rate. The identified differentially expressed genes underwent Reactome analyses using the cluster profile package, and significance was attributed to an adjusted *p*-value of < 0.05. The GSEA and iDEP were also employed to elucidate the pathways differentially enriched between groups [30, 31].

### GeoMx DSP whole transcriptome and protein nCounter workflow

Liver Sect. (4 μm) of healthy, HCC, MAFLD, and MASH-HCC mice were used for digital spatial profiler (DSP) of whole transcriptome sequencing (NanoString, WA, USA). The panel of morphology markers used were CD45, SYTO13 nuclear stain, and Pan-cytokeratin. RNAscope probes and GeoMx DSP oligo-conjugated RNA detection probes were used to stain the sections following the manufacturer’s protocol [26]. Twelve regions of interest (ROIs) were chosen, each with a diameter of 300–600 μm per group (inside the tumors, at the tumor margin, and outside the tumor, 4 per location). The FASTQ sequencing files were converted into digital count files using Nanostring’s GeoMx NGS Pipeline software. Quality control and data analysis were performed using the GeoMx DSP Data Analysis suite. The data were filtered by the limit of quantitation and then normalized by the third quartile of all counts.

A DSP Protein Immune Cell Profiling Panel (NanoString, WA, USA) consists of PD1, PD-L1, MHCII, CD11b, Ki67, CD11c, CD19, CD3e, CD4, CTLA4, and GZMB. The panel was used to study their protein level spatially using a nCounter-based readout based on the manufacturer’s protocol. Fifteen regions of interest (ROIs) per HCC and MASH-HCC (inside the tumors, at the tumor margin, and outside the tumor, 5 per location) were chosen. The GeoMx data underwent normalization based on control molecules, including housekeeping proteins and isotype controls.

### Cell deconvolution analyses

The GeoMx DSP control center utilized the spatialdecon geoscript (v1.3, updated October 2022) from Nanostring’s Geoscript Hub to conduct cell deconvolution analyses. [https://nanostring.com/products/geomx-digital-spatial-profiler/geoscript-hub/].

### Bioinformatics analysis and comparison with human MASH-HCC

The immune cell abundance was determined by the enrichment score of the expression deviation profile per cell type using single-sample gene set enrichment analysis (ssGSEA) [32, 33]. The obtained enrichment score was normalized, resulting in the ultimate immune cell abundance (http://bioinfo.life.hust.edu.cn/web/ImmuCellAI/).

The normalized read counts of the human MASH-HCC, previously known as NASH-HCC (*n* = 53), and healthy human liver controls (*n* = 6) were obtained from the GSE164760 dataset, which is available in the Gene Expression Omnibus in NCBI [34]. We analyzed the data using the Reactome [35]. The significant enrichment was considered at FDR < 25%.

### Statistical analysis

Quantitative data were presented as means ± standard deviation (SD). Group comparisons were conducted using one-way ANOVA followed by a Tukey test. The Mann–Whitney test was employed to compare the two groups. Statistical analyses were performed using GraphPad Prism 10.0 software (San Diego, CA), and significance was determined at *p* values < 0.05.

## Result

### Morphological characteristics of MASH-HCC

**Fig. 1:**
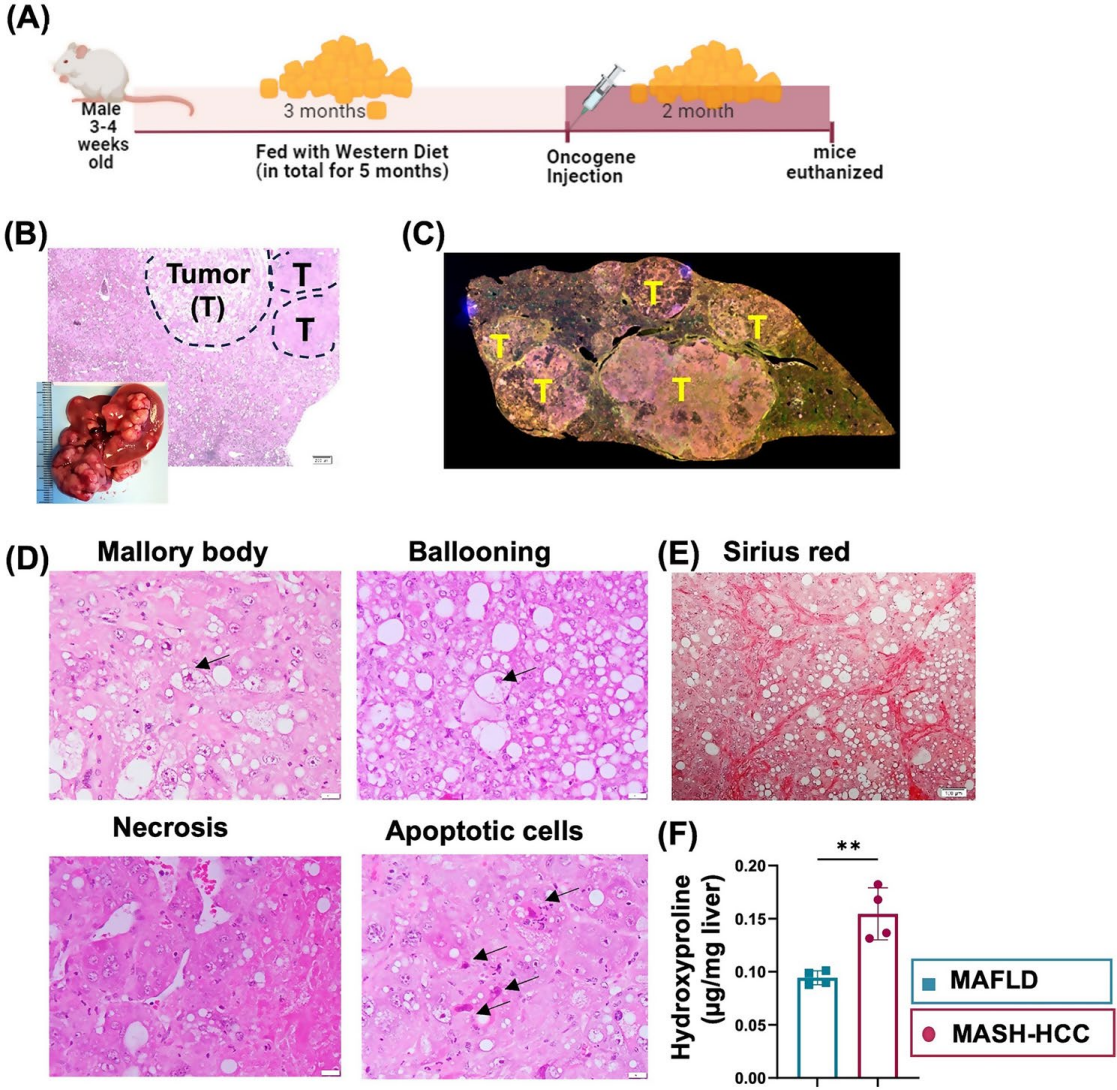
Characteristics of MASH-HCC. (**A**) Experimental design: To induce MASH-HCC, after three months of Western diet (WD) intake, plasmids myr-Akt1 and N-RasV12 were injected to induce HCC. The mice were continued a WD for an additional two months before being euthanized. MAFLD was induced by feeding mice WD for five months. (**B**) Representative liver gross morphology and H&E-stained liver sections (magnification 4x). (**C**) A liver section showing tumor (T) occupied areas. (**D**) MASH-HCC is featured with Mallory’s body, ballooning, necrosis, and apoptotic cells. (**E**) Sirus red staining visualized the presence of collagen fibers in MASH-HCC. (**F**) Hydroxyproline assay was used to quantify the hepatic collagen concentration of MAFLD and MASH-HCC. Data were shown as mean ± SD (*n* = 6/group). *T-Test* was used; **, *p* < 0.01

The experimental design is summarized in Fig. 1A. The gross morphology revealed the presence of tumors, supported by histology (Fig. 1B, C). Outside the tumors, the livers were featured with steatosis, the presence of Mallory bodies, necrosis, ballooning, and apoptotic cells (Fig. 1D). Sirius red staining revealed fibrosis (Fig. 1E). Additionally, hydroxyproline assays showed that MASH-HCC had higher hepatic collagen concentration than WD-induced MAFLD even though both groups had the same duration of WD intake, i.e., five months (Fig. 1F).

### The molecular signatures affected by the Western diet in healthy livers and HCC

**Fig. 2:**
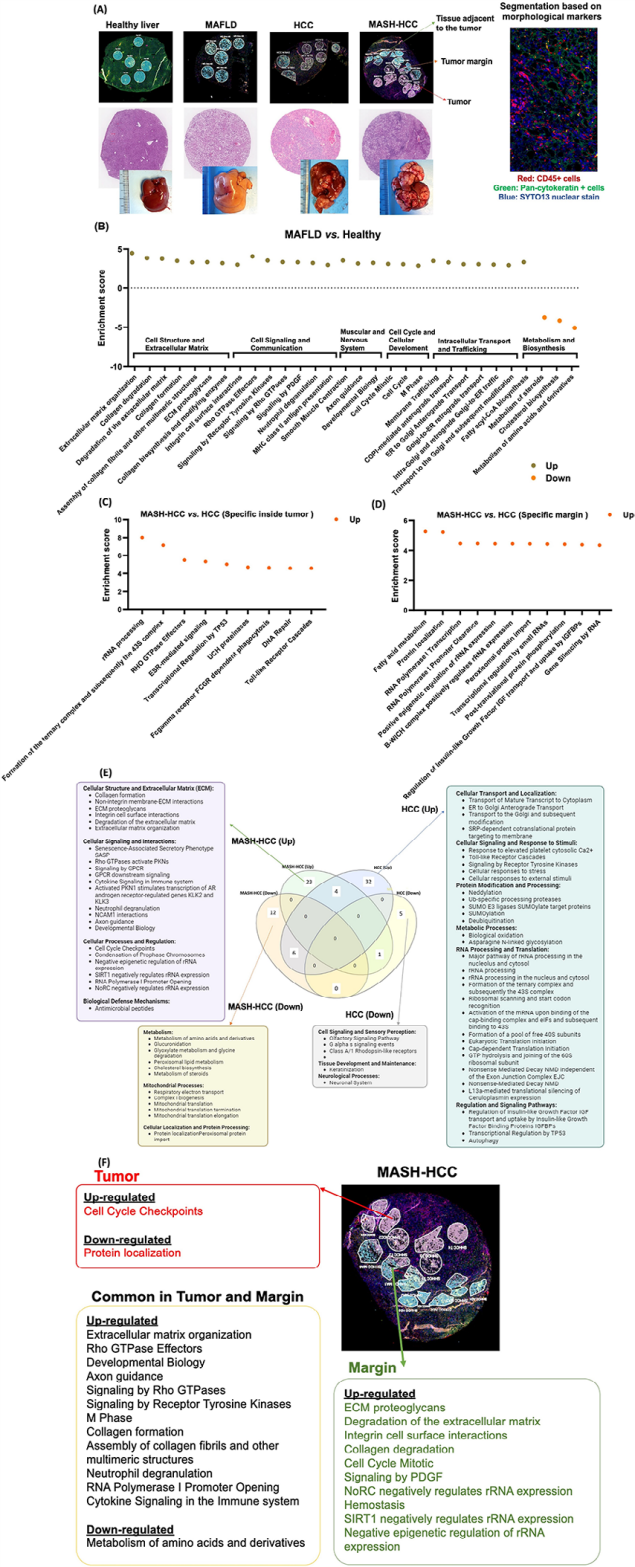
The molecular signatures affected by Western diet intake. (**A**) Representative images of region of interest (ROI), gross and histology images, and segmentation based on morphological markers. (**B**) Pathways that are significantly altered in MAFLD compared with healthy livers. (**C**) Pathways significantly enriched in MASH-HCC vs. HCC inside the tumors, identified through spatial RNA transcriptomics. (**D**) Pathways that are significantly enriched in MASH-HCC vs. HCC in margin based on spatial RNA transcriptomics (**E**) Pathways that were specifically enriched in MASH-HCC (left box) or HCC (right box) compared to healthy liver using bulk RAN sequencing. (**F**) Pathways enriched in MASH-HCC were identified through bulk transcriptomic analysis and aligned with spatial transcriptomics data to map the location of uniquely enriched pathways in MASH-HCC. All pathways were analyzed using Reactome

The gross and spatial microscopy images of a healthy liver, MAFLD, HCC, and MASH-HCC are shown in Fig. 2A. Figure 2A also provides an example of selecting a region of interest (ROI) and further segmentation to select the area of interest illumination (AOI) based on CD45 (red) or pan-cytokeratin (green) and SYTO13 nuclear stain (blue) markers. We first studied the impact of WD in the livers without HCC. Transcriptomic analysis revealed WD intake significantly enriched ECM, collagen formation, and degradation, as well as integrin cell surface interaction accompanied by heightened Rho and platelet-derived growth factor (PDGF) signaling (Fig. 2B). The Rho family of GTPases have crucial roles in cytoskeletal dynamics, cell migration, cell adhesion, and cell proliferation, thereby involving the formation of stress fibers, focal adhesions, and other cytoskeletal structures [36]. Moreover, WD intake amplified neutrophil degranulation and MHC class II antigen presentation, revealing elevated immune response accompanied by increased cell cycle, M phase, and mitosis. Furthermore, fatty acyl-CoA biosynthesis was enriched, while the metabolism of steroids and nutrients was downregulated in MAFLD (Fig. 2B).

To uncover the impact of WD in liver tumorigenesis, MASH-HCC was directly compared to HCC at the spatial transcriptomics level (Fig. 2C). Pathways related to translation, RNA processing and decay, cellular responses, cell cycle, and senescence were upregulated in MASH-HCC compared to HCC, in all locations (Supplemental Table 1). However, signaling related to GTPase effector, phagocytosis, and DNA repair upregulated specifically inside the tumor when MASH-HCC compared to HCC. In the margin, pathways related to metabolism, protein localization RNA transcription and processing, and regulation of growth factors were upregulated in MASH-HCC (Fig. 2D).

To uncover tumor-specific pathways, bulk transcriptomics or MASH-HCC and HCC were compared with the healthy livers (Figure E). A Venn diagram illustrates the common and distinctive pathways that were either upregulated or downregulated in MASH-HCC or HCC after comparing with healthy livers (Figure E). MASH-HCC had marked enrichment of ECM, cytokine signaling in the immune system, and senescence-associated secretory phenotype (SASP) accompanied by upregulation of cell cycle checkpoints. Interestingly, the anti-microbial peptide pathway was upregulated (Fig. 2E, left box). In contrast, when HCC was grown in a healthy liver, the uniquely upregulated pathways were cellular transport, cellular signaling in response to stimuli, protein modification, processing, metabolic processes, and RNA processing (Fig. 2E, right box). The commonly upregulated pathways in MASH-HCC and HCC (at the margin and inside the tumor) were Rho GTPase effectors, signaling by Rho GTPases, M phase, and cell cycle mitotic. In addition, there were pathways enriched in both MASH-HCC and HCC, but in opposite directions. For instance, citric and TCA cycle, ATP synthesis, and translation (at the margin and inside of the HCC and MASH-HCC tumors) were upregulated in HCC but downregulated in MASH-HCC. Interestingly, GPCR (G protein-coupled receptor) ligand binding was inhibited in MASH-HCC but upregulated in HCC. Because many intestinal bacterial metabolites, such as short-chain fatty acids, can bind to GPCR, the data suggests that diet-gut microbiomes influence the liver tumor environment.

The pathways enriched in MASH-HCC using bulk transcriptomic data were aligned with spatial transcriptomic data to map the location. As anticipated, upregulation of cell cycle checkpoints was found inside the tumor (Fig. 2F, red). Tumor also had downregulation in protein localization. Upregulation of ECM proteoglycans, degradation of the matrix and collagen, integrin cell surface interaction, and PDGF signaling were found at the tumor margin (Fig. 2F, green). Moreover, pathways related to ECM organization, Rho signaling, immune response, cytokine signaling, and cell cycle regulation were upregulated inside the tumor and at the tumor margin (Fig. 2F, in black).

Due to the significance of the WD in inducing inflammation, the top 40 inflammatory genes upregulated in MASH-HCC in comparison with healthy livers are summarized in a heatmap. Genes implicated in IL17, TNF, IL1β, IL33, IL34, etc. signaling were markedly induced in MASH-HCC (Supplemental Fig. 2).

### Enriched ECM and EMT signaling in mouse and human MASH-HCC

**Fig. 3:**
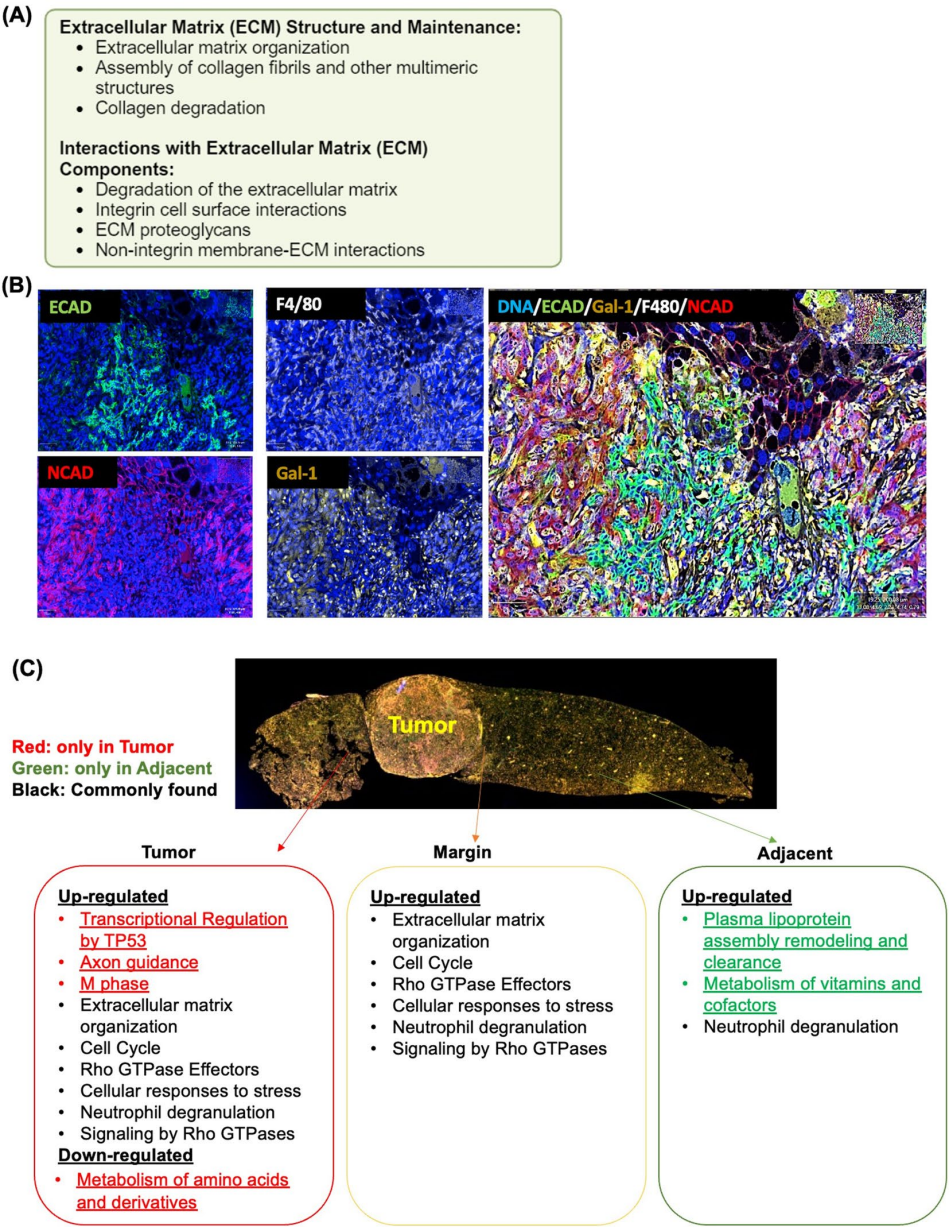
Molecular signatures of human and mouse MASH-HCC. (**A**) Common pathways that are enriched in human and mouse MASH-HCC compared with their respective healthy livers using bulk RNA sequencing. (**B**) The multiplex immunohistochemistry (IHC) staining of E-cadherin (ECAD), N-cadherin (NCAD), F4/80, and Gal-1 in mouse MASH-HCC. (**C**) Assigning human MASH-HCC bulk transcriptomic data spatially to align with mouse data. Human MASH-HCC bulk RNA sequencing data were from GSE164760, available in the NCBI [34]. The human data comprises healthy livers (*n* = 6) and MASH-HCC (*n* = 53). For mice, the sample size was 4 per group (healthy livers, HCC, and MASH-HCC)

Even in the livers without HCC, our data point to the significance of WD in heightening ECM. To determine human relevance, bulk RNA sequencing data from human steatohepatitis HCC (GSE164760) were compared with mouse MASH-HCC. The ECM structure and maintenance and interaction with ECM components were noted in humans and mice (Fig. 3A). Significant upregulation in pathways consisted of ECM organization, degradation, integrin cell surface interactions, ECM proteoglycans, non-integrin membrane-ECM interactions, assembly of collagen fibrils, and collagen degradation.

Given the substantial enrichment of the ECM organization pathways in human and mouse models of MASH-HCC, multiplex-IHC was performed to study critical molecules involved in EMT. Those included ECAD, NCAD, and Gal-1. Gal-1 is a critical player in inducing EMT by increasing NCAD but reducing ECAD, and it has known immune suppressive roles [37, 38]. Moreover, Gal-1 also interacts with Rho GTPases, and laminin, thereby affecting matrix remodeling [39]. F4/80 and Syto13 were used to identify macrophages and DNA, respectively (Fig. 3B). The data revealed that the NCAD (red)-enriched area had much more Gal-1-positive cells (brown) and macrophages (white) in contrast to the ECAD (green)-positive area. Those findings further revealed the significance of a WD in enhancing Gal-1-regulated EMT signaling and macrophage infiltration, thus altering the tumor environment.

To our knowledge, no human MASH-HCC spatial transcriptomic data is available in the public domain. Therefore, we assigned human MASH-HCC bulk transcriptomic data spatially to align with mouse data. Upregulated TP53-regulated transcription, axon guidance, and M phase, as well as downregulated metabolism of amino acids and derivatives, were mapped inside the tumor (Fig. 3C, red). However, upregulated plasma lipoprotein assembly and clearance, as well as the metabolism of vitamins, were only mapped in non-tumorous tissues adjacent to the tumor (Fig. 3C, green). Both tumor and margin had upregulated ECM organization, Rho GTPase effectors, signaling by Rho GTPases, cellular response to stress, neutrophil degranulation, and cell cycle signaling (Fig. 3C, black). Notably, neutrophil degranulation was upregulated in all three locations.

### Spatial immune profiles of MASH-HCC vs. HCC

The relative abundance of immune cells in the liver sections was estimated and compared in MASH-HCC and HCC using an immune cell abundance identifier (ImmuCellAI-mouse).

**Fig. 4:**
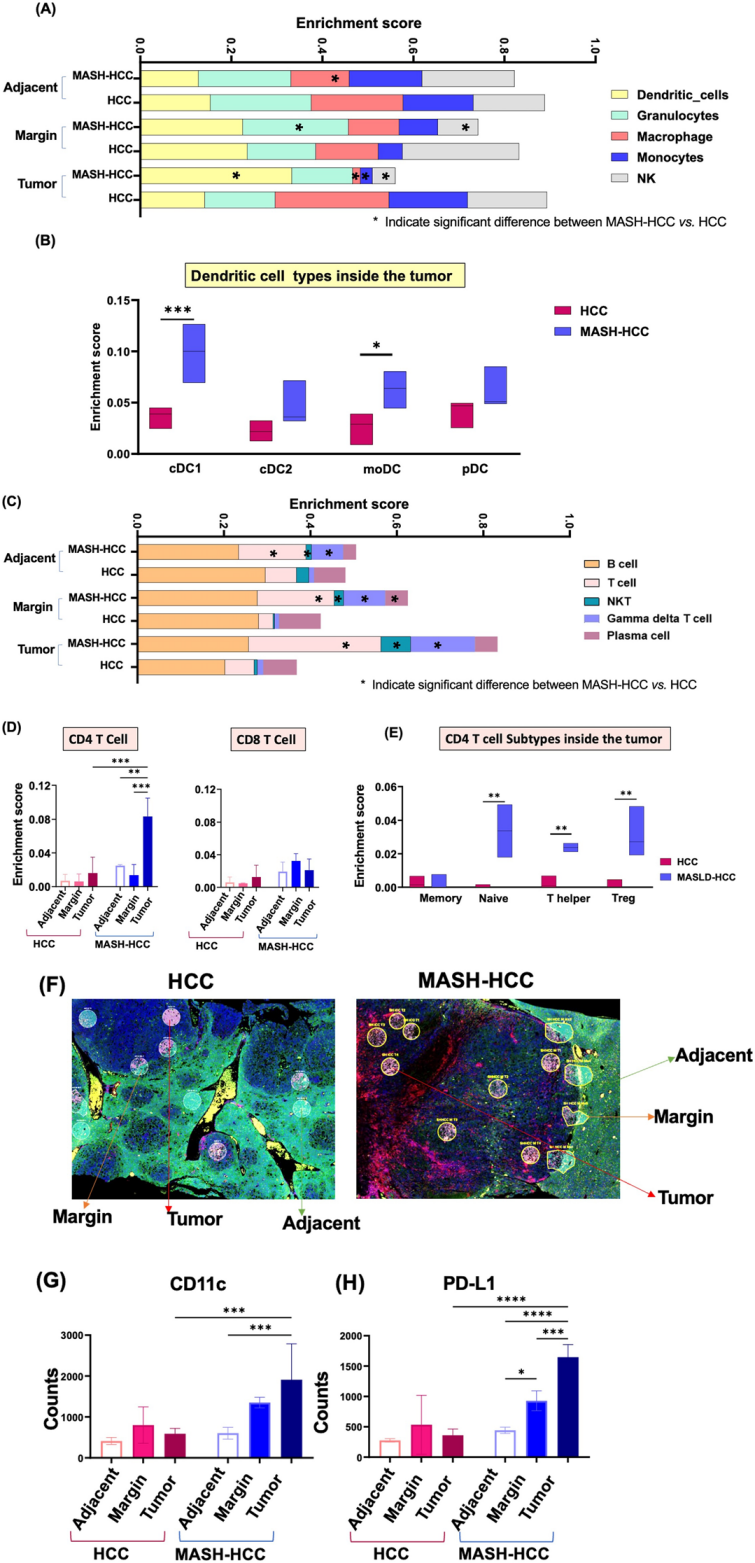
Spatial immune profiles of MASH-HCC and HCC. **(A and C)** Immune profiling of CD45 + cells in the adjacent nontumor areas, tumor margin, and inside the tumors of HCC and MASH-HCC mice. Stars indicate the significance between MASH-HCC vs. HCC in each location. (**B**) Dendritic cell subtypes: conventional dendritic cells (cDC1 and cDC2), monocyte-derived dendritic cells (moDCs), and plasmacytoid dendritic cells (pDCs). **(D)** Relative abundance of CD4 and CD8 T cells based on the location. (**E**) CD4 T cell subtypes: naïve CD4 T cell, T helper, and Treg inside the tumors. (**F**) The example of nCounter is from selecting the region of interest (ROI) based on location. (**G**) The protein counts of CD11c and PD-L1-positive cells in each location in HCC and MASH-HCC mice. Data shown are mean ± SD. ANOVA-Tukey was used; *, *p* = 0.05**, *p* = 0.01, ***, *p* = 0.001

In non-tumorous adjacent tissues, macrophages were significantly lower in MASH-HCC compared to HCC. At the tumor margin, MASH-HCC had much fewer natural killer (NK) cells but more granulocytes relative to HCC. Inside the tumor, MASH-HCC had fewer monocytes, macrophages, and NK cells but a higher count of dendritic cells compared to HCC (Fig. 4A). Further analyses of dendritic cell subtypes indicated a significant increase in conventional dendritic cells (cDC1) and monocytes-derived dendritic cells (moDCs) inside the tumors in MASH-HCC compared to HCC (Fig. 4B). No significant differences were observed in cDC2 and plasmacytoid dendritic cells (pDCs) between MASH-HCC and HCC (Fig. 4B).

Interestingly, γδ T cells were consistently enriched in all three locations in MASH-HCC than in HCC. Natural killer T cells (NKT), which bridge the innate and adaptive immunity [40], were reduced in non-tumorous adjacent tissues but increased in the margin and tumors of MASH-HCC (Fig. 4C). No significant difference was noted for B cells. The reduction of plasma cells in MASH-HCC was noted at the margin. Furthermore, CD4 + T cells, but not CD8 + T cells, were significantly increased inside the MASH-HCC tumor (Fig. 4D). Further analysis of CD4 + T cells in the tumor revealed that naïve CD4, T helper, and Treg cells were significantly higher in MASH-HCC relative to HCC (Fig. 4E). Those changes likely influence the tumor microenvironment. An increased number of naïve CD4 + T helper cells may lead to enhanced immune surveillance and potential anti-tumor responses once these cells differentiate into functional T helper subsets. However, the elevated Treg cells could contribute to an immunosuppressive environment, inhibiting effective anti-tumor responses and potentially facilitating tumor progression. The combined increase in these CD4 + T cell subsets may lead to a complex and often conflicting immune environment.

We further studied immune cell markers at the protein level using nCounter-Nanostring technology (Fig. 4F). Among the studied proteins, including PD1, PD-L1, MHCII, CD11b, Ki67, CD11c, CD19, CD3e, CD4, CTLA4, and GZMB, the most distinctive changes were noted for CD11c and PD-L1. CD11c, also known as integrin alpha X, is a widely used marker for dendritic cells as well as inflammatory monocytes; it plays a crucial role in cell-cell and cell-matrix interactions [41]. PD-L1, by binding to PD-1, inhibits T cell activation, proliferation, and survival to escape immune surveillance [42]. While HCC did not show changes in expression levels of CD11c and PD-L1 based on the location, MASH-HCC had progressively increased expression of both from non-tumorous adjacent tissues, margin, to inside tumor (Fig. 4G and H). Thus, WD creates an immune-tolerant environment based on the location.

### The spatial effects of Gal-1

As MASH-HCC is featured with overexpressed Gal-1 accompanied by augmented matrix remodeling and EMT, we studied the spatial effects of inhibiting Gal-1 in MASH-HCC. The Gal-1 silencing (*lgals1* siRNA) construct was delivered one month after tumor initiation, and the mice were euthanized one month later. Gal-1 silencing markedly reduced the tumor burden. While the control (AAV9-treated) MASH-HCC mice had a mean L/B of 18%, the L/B of AAV9- *lgals1* siRNA-treated mice was only 7.5%, not different from that of MAFLD mice (bar graph not shown).

Spatially, transcriptomics data revealed that signaling related to immune and ECM regulation, such as the adaptive immune system and degradation of the ECM, as well as cell adhesion, like neural cell adhesion molecule (NCAM) signaling for neurite outgrowth, were among the most affected signaling downregulated due to Gal-1 silencing found at the tumor margin (Supplemental Fig. 2A). Remarkably, the adaptive immune system was the most downregulated pathway, suggesting a reduced immune response at the margin when the tumor shrunk.

**Fig. 5:**
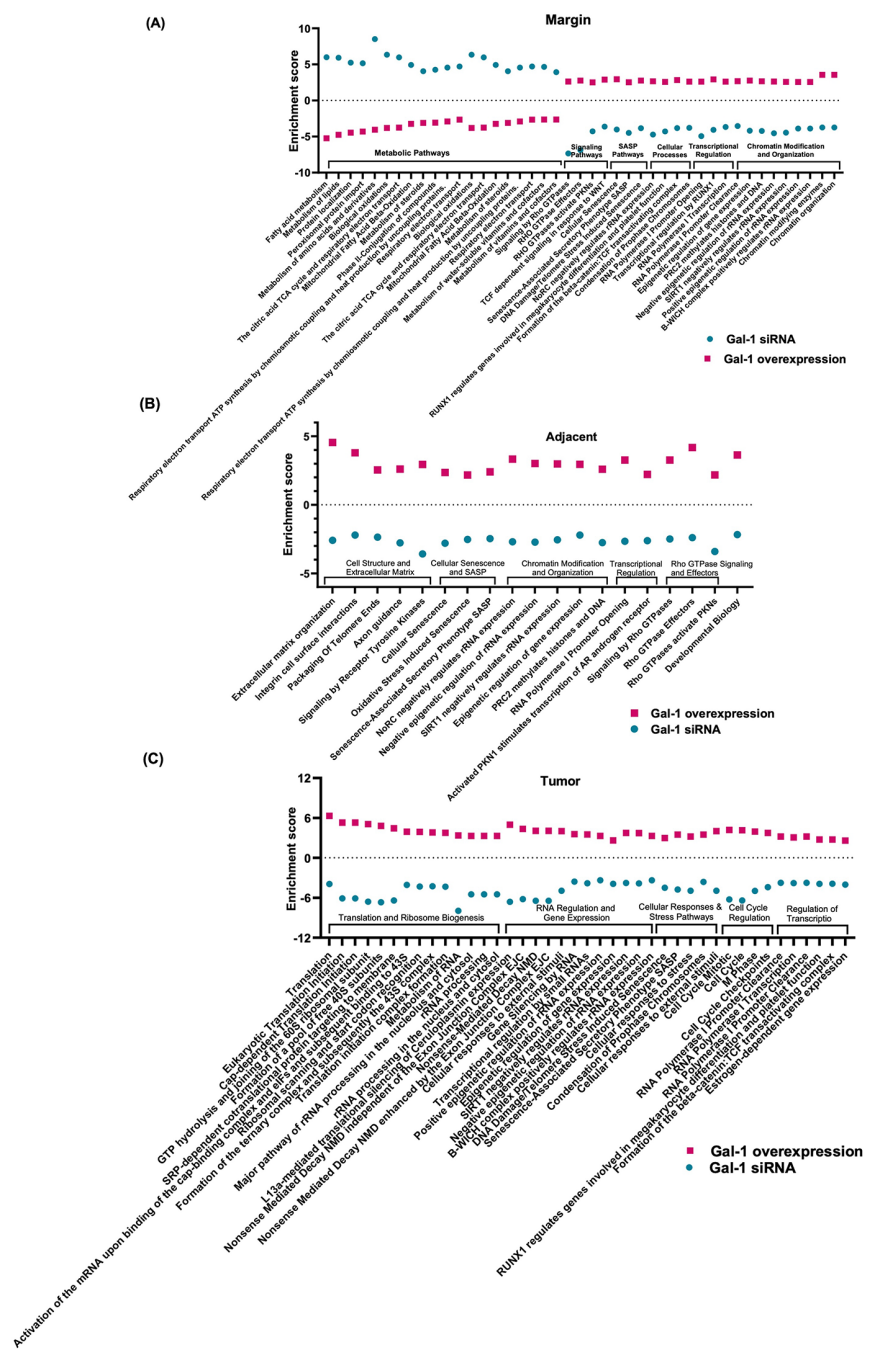
Pathways that are regulated in opposite directions due to Gal-1 silencing and overexpression in MASH-HCC. Common pathways (based on Reactome) that were significantly (FDR cutoff: 0.1 and min fold change of 1.5) altered in opposite directions due to Gal-1 overexpression or silencing in MASH-HCC; (**A**) tumor margin, (**B**) adjacent tissues, and (**C**) inside the tumor

We further studied whether Gal-1 overexpression would affect matrix and EMT pathways at the same location. Thus, forced expression of Gal-1 was done by delivery of AAV9-Gal-1 one month before the MASH-HCC mice were euthanized. At the margin, signaling pathways (i.e., Rho GTPases, Rho GTPase effectors, and Rho GTPases activate) showed the greatest reduction by Gal-1 silencing but an increase by Gal-1 overexpression, signifying Gal-1 specific effects (Fig. 5A). Other Gal-1 specific pathways at the tumor margin included SASP and DNA damage-related pathways (i.e., cellular senescence, DNA damage/telomere stress-induced senescence), cellular process (i.e., formation of β-catenin) as well as transcriptional regulation and chromatin modification (Fig. 5A). In contrast, metabolic pathways (i.e., fatty acid, lipid, amino acid metabolisms, TCA cycle, mitochondria fatty acid β-oxidation) were significantly upregulated by Gal-1 silencing but downregulated by Gal-1 overexpression revealing improved liver function at the margin (Fig. 5A).

In the adjacent non-tumor area, silencing Gal-1 downregulated ECM interaction and adhesion, immune regulation, signaling pathways related to platelet degranulation and Rho GTPase activate NADPH oxidase (Supplemental Fig. 2B). Gal-1-specific signaling was also identified at the adjacent area. These pathways were commonly found in Gal-1 silencing and overexpression but were regulated in opposite directions, which included cell structure and ECM organization, cellular senescence, SASP, Rho GTPase signaling, etc. (Fig. 5B).

Inside the tumor, silencing Gal-1 downregulated cell cycle, RNA processing and transcription regulation, and cell signaling and regulation (Supplemental Fig. 2C). Many Gal-1-specific pathways were found inside the tumors (i.e., pathways related to translation and ribosome biogenesis, RNA regulation, and gene expression and regulation of transcription) (Fig. 5C). Thus, Gal-1 modified the SASP and DNA damage/telomere senescence pathways in MASH-HCC in all three locations (Fig. 5C).

### WD affects gal-1-mediated signaling for tumorigenesis

**Fig. 6:**
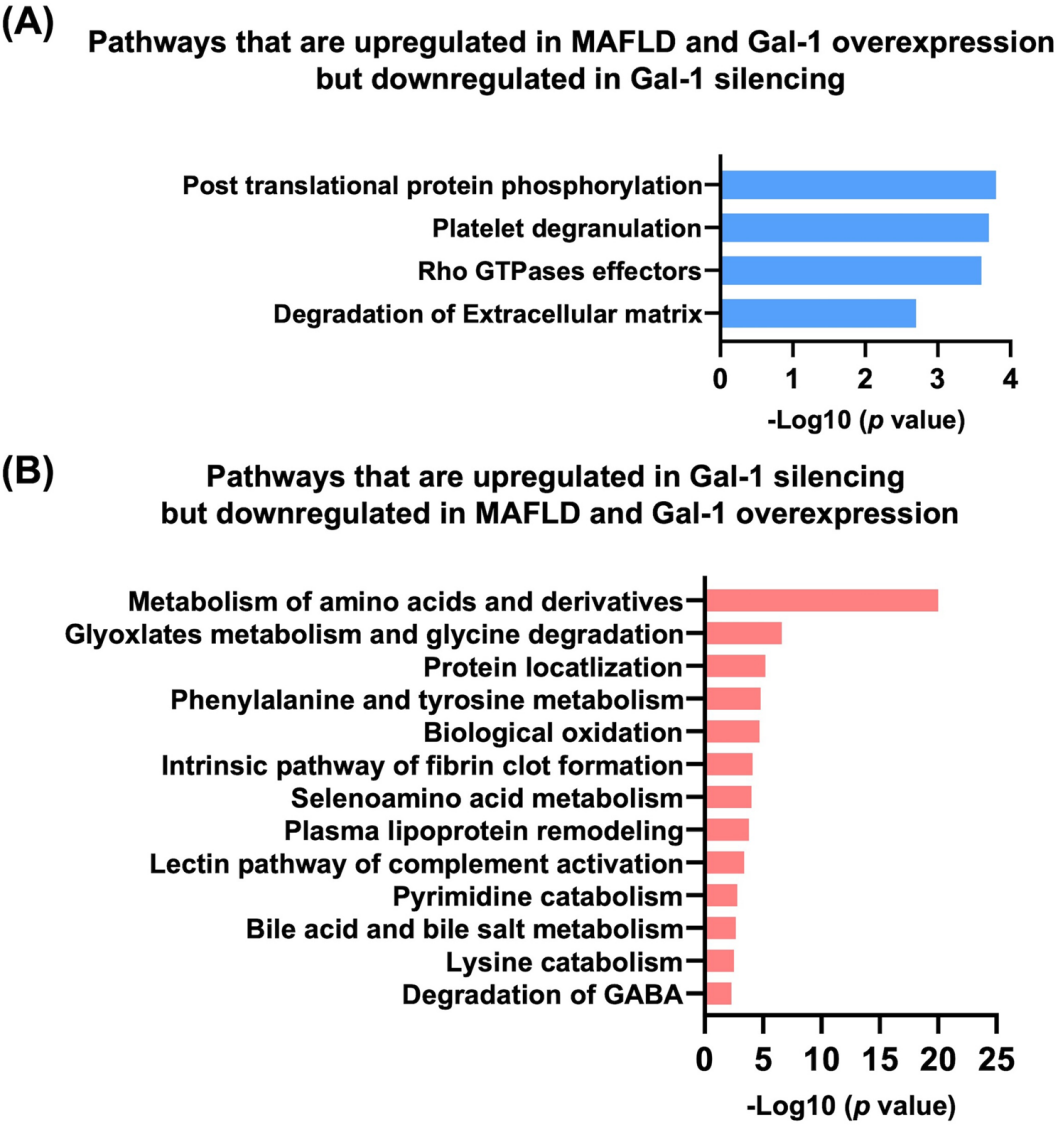
The Western diet regulates Gal-1-mediated signaling (**A**) Pathways that were upregulated in MAFLD and Gal-1 overexpression but downregulated in Gal-1 silencing (**B**) Pathways that were upregulated in Gal-1 silencing but downregulated in MAFLD and Gal-1 overexpression (Based on Reactome)

We further analyzed the common effects of a WD and Gal-1 using bulk RNA sequencing data. Post-translational phosphorylation (*Sparc11*), platelet degranulation (*Gas6*), Rho GTPase effectors (*Spp1*), and degradation of ECM were commonly upregulated by WD intake and Gal-1 overexpression but downregulated by Gal-1 silencing (Fig. 6A). Moreover, the metabolism of amino acids and derivatives (i.e.,* Sardh*,* Fah*,* Agmat*,* Aldh4a1*), protein localization, bile acid metabolism, and degradation of GABA were among the upregulated pathways due to Gal-1 silencing. However, those pathways were downregulated by WD intake and Gal-1 overexpression (Fig. 6B).

## Discussion

This study might be one of the first to characterize the molecular landscape of a novel MASH-HCC using genome-wide spatial transcriptomics providing two-dimensional positional information in liver sections. Using both healthy liver and HCC models, the impact of a WD is uncovered using non-biased approaches. In mice without HCC, WD intake heightened many cellular signaling pathways that crosstalk. For instance, WD-induced PDGF stimulates integrin activation, affecting cell migration and proliferation. Integrins, in turn, can modulate PDGF-mediated responses [43]. PDGF also activates Rho GTPases, influencing the actin cytoskeleton and cell migration [44]. Moreover, integrins can be activated by growth factors, such as those binding to receptor tyrosine kinase, which is also upregulated in MAFLD mice. Further, receptor tyrosine kinase activation can lead to Rho GTPase activation, coordinating growth factor-mediated signaling with cytoskeletal dynamics [45]. Signaling of receptor tyrosine kinase can also influence the activation and maturation of antigen-presenting cells, impacting the efficiency of MHC class II antigen presentation to T cells. These interactions illustrate coordinated responses to WD intake affecting immune responses and matrix remodeling, thereby creating a tumor-supporting environment.

Compared with HCC produced in healthy liver without MASH, heightened Rho, ECM, and EMT signaling pathways were also found in MASH-HCC. Moreover, MASH-HCC had augmented cytokine signaling and SASP. These findings are consistent with our recent bioinformatic data that showed WD intake facilitates liver aging [46]. Among the cytokines signaling pathways, the expression levels of genes related to TNFR and TNF signaling as well as the *Il6*, *Il17*, and *Il-1β* cytokine family, were uniquely enriched in MASH-HCC (Supplementary Fig. 1).

Examining immune cell profiles in MASH-HCC vs. HCC, inside the tumor, MASH-HCC had much more dendritic cells but fewer macrophages, monocytes, and NK cells. NK cells can directly kill cancer cells without prior sensitization; their reduced presence in MASH-HCC may hinder immune surveillance against tumor cells. Additional analysis unveiled that cDC1 and moDC were increased in MASH-HCC relative to HCC. cDC, typically classified as CD103+, can induce the differentiation of Tregs [47]. Tregs, in turn, exert immunosuppressive effects, potentially modulating the immune response within the tumor milieu. In consistency, the amount of Treg was much more inside the tumor of MASH-HCC than that of HCC. moDCs are highly effective at capturing, processing, and presenting antigens to T cells, suggesting a highly active inflammatory environment in MASH-HCC. Increased γδ T cells were consistently found in all three locations of MASH-HCC compared with those of HCC. γδ T cells have many unique functions ranging from recognition of a diverse range of antigens, including lipids, phospholipids, and small organic molecules, without the need for antigen processing and presentation by MHC molecules [48]. Furthermore, they can produce IL-17 and interferon γ, influencing the polarization of immune responses [49, 50]. As mentioned above, short-term intake of WD induces IL-17 signaling [7]. In parallel, increased γδ T cells were accompanied by increased IL-17 receptor (*Il17r*) and its coreceptor (*Il17rd*), shown in Supplemental Fig. 1.

Analyzing immune cell markers at the protein level revealed the expansion of CD11c and PD-L1 cells inside the tumor. CD11c (integrin αX) is a receptor of ECM and a biomarker of dendritic cells [51]. The expansion of CD11c-positive cells inside the tumor of MASH-HCC was consistent with the findings that MASH-HCC had increased DCs and ECM, particularly inside the tumor, as revealed by analyzing transcriptomic data. The progressive increase of CD11c and PD-L1 cells from non-tumorous tissues to tumors in MASH-HCC suggests an interaction between increased DCs and the immunosuppressive PD-L1. Thus, an immunosuppressive tumor environment is augmented in MASH-HCC [52, 53].

The data generated using Gal-1 silencing and overexpression provided solid findings for the spatial effects of Gal-1 in regulating Rho and matrix signaling in the tumor margin and adjacent to the tumor. The Rho family of GTPases is a family of G proteins and part of the RAS superfamily. However, no oncogenic mutations have been found in Rho proteins. One of the major roles of the Rho proteins is to regulate the actin cytoskeleton involved in cellular processes like cell migration, polarity, or movement [54]. The signaling transduction *via* Rho GTPases is implicated in the progression of cancer [55]. The current study revealed that Gal-1 likely regulates Rho signaling at multiple levels, covering Rho GTPases, effectors, and GTPases that activate protein kinase. Moreover, the coordinated changes of Rho and matrix signaling signifies the significance of Gal-1 *via* Rho signaling to impact matrix remodeling and EMT.

## Conclusions

The current study reveals the extensive impact of a WD in influencing HCC tumor environment. The represented data supports the effects of a WD in stimulating Gal-1-mediated signaling, ranging from Rho, matrix remodeling, to EMT. All those changes affect carcinogenesis and tumor immunity. Thus, a WD not only facilitates liver carcinogenesis but also likely impacts HCC immunotherapy outcomes. It would be interesting to further compare the effects of a WD vs. HFD on tumor microenvironment.

## Electronic supplementary material

Below is the link to the electronic supplementary material.

Supplementary Material 1: Supplemental Table 1. Supplementary Table 1. Common pathways that are enriched in inside tumor and at the margin in MASH-HCC and HCC.

Supplementary Material 2: Supplemental Fig. 1. Top 40 genes that are significantly upregulated in MASH-HCC compared with healthy livers. FDR cut off 0.1 and min fold change 1.5.

Supplementary Material 3: Supplemental Fig. 2. Pathways that are regulated by Gal-1 silencing in MASH-HCC. (**A**) tumor margin, (**B**) adjacent tissues, and (**C**) inside the tumor. Based on Reactome

## Data Availability

The datasets used and/or analyzed during the current study are available from the corresponding author upon reasonable request.

## Abbreviations

AAV9: Adeno-associated virus serotype 9
cDC: Conventional dendritic cells
DSP: Digital spatial profiler
ECAD: E-cadherin
ECM: Extracellular Matrix
EMT: Endothelial to mesenchymal transition
Gal-1: Galectin-1
γδT cell: Gamma-delta T cell
GPCR: G protein-coupled receptor
HCC: Hepatocellular carcinoma
H&E: Hematoxylin, and eosin
HFD: High-fat diet
IHC: Immunohistochemistry
IL: Interleukin
MAFLD: Metabolic-associated fatty liver disease
MASH: Metabolic dysfunction-associated steatohepatitis
moDCs: monocytes-derived dendritic cells
NCAD: N-cadherin
NK: Natural Killer
pDCs: plasmacytoid dendritic cells
PDGF: Platelet-derived growth factor
ROIs: Region of interest
SASP: Senescence-associated secretory phenotype
WD: Western diet
