# Supplementary figures and images for: The spatial impact of a Western diet in enriching Galectin-1-regulated Rho, ECM, and SASP signaling in a novel MASH-HCC mouse model
Source: Biomark Res. 2024 Oct 14;12:122. doi: 10.1186/s40364-024-00660-3 (PMC11476289; doi:10.1186/s40364-024-00660-3)

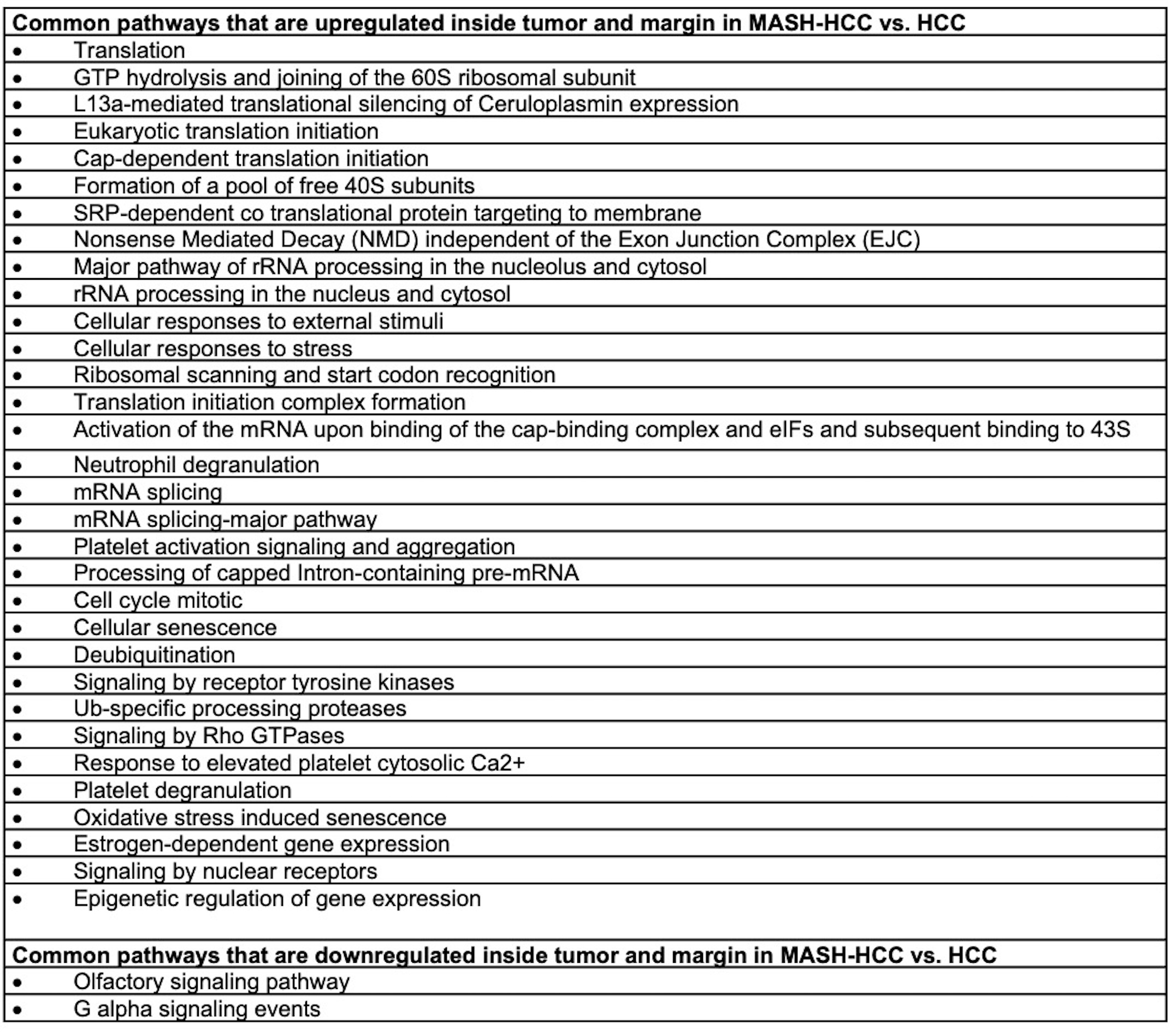

Supplement: Supplementary file 1 — Supplementary Material 1: Supplemental Table 1. Supplementary Table 1. Common pathways that are enriched in inside tumor and at the margin in MASH-HCC and HCC. [file 40364_2024_660_MOESM1_ESM.jpg]

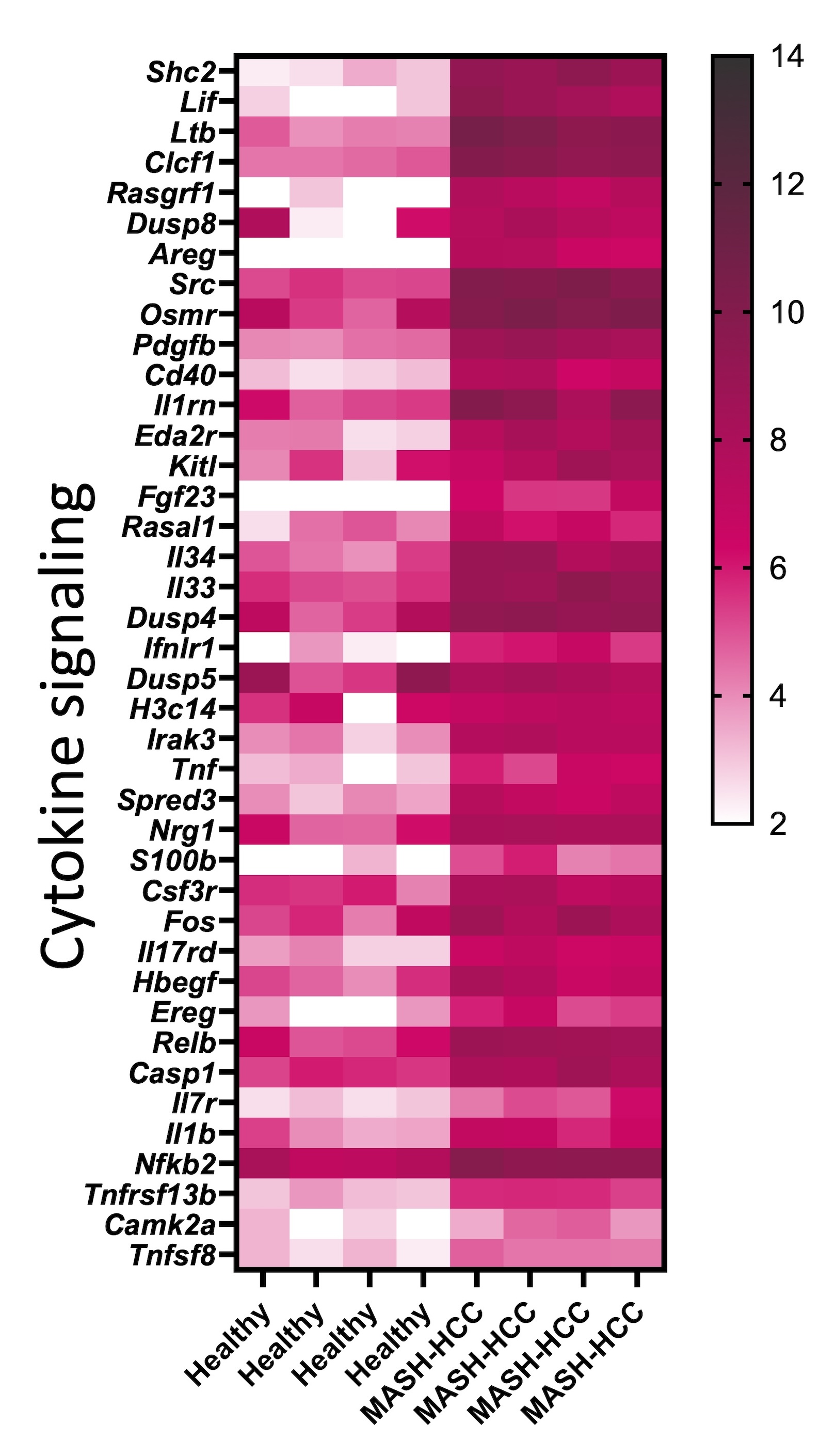

Supplement: Supplementary file 2 — Supplementary Material 2: Supplemental Fig. 1. Top 40 genes that are significantly upregulated in MASH-HCC compared with healthy livers. FDR cut off 0.1 and min fold change 1.5. [file 40364_2024_660_MOESM2_ESM.jpg]

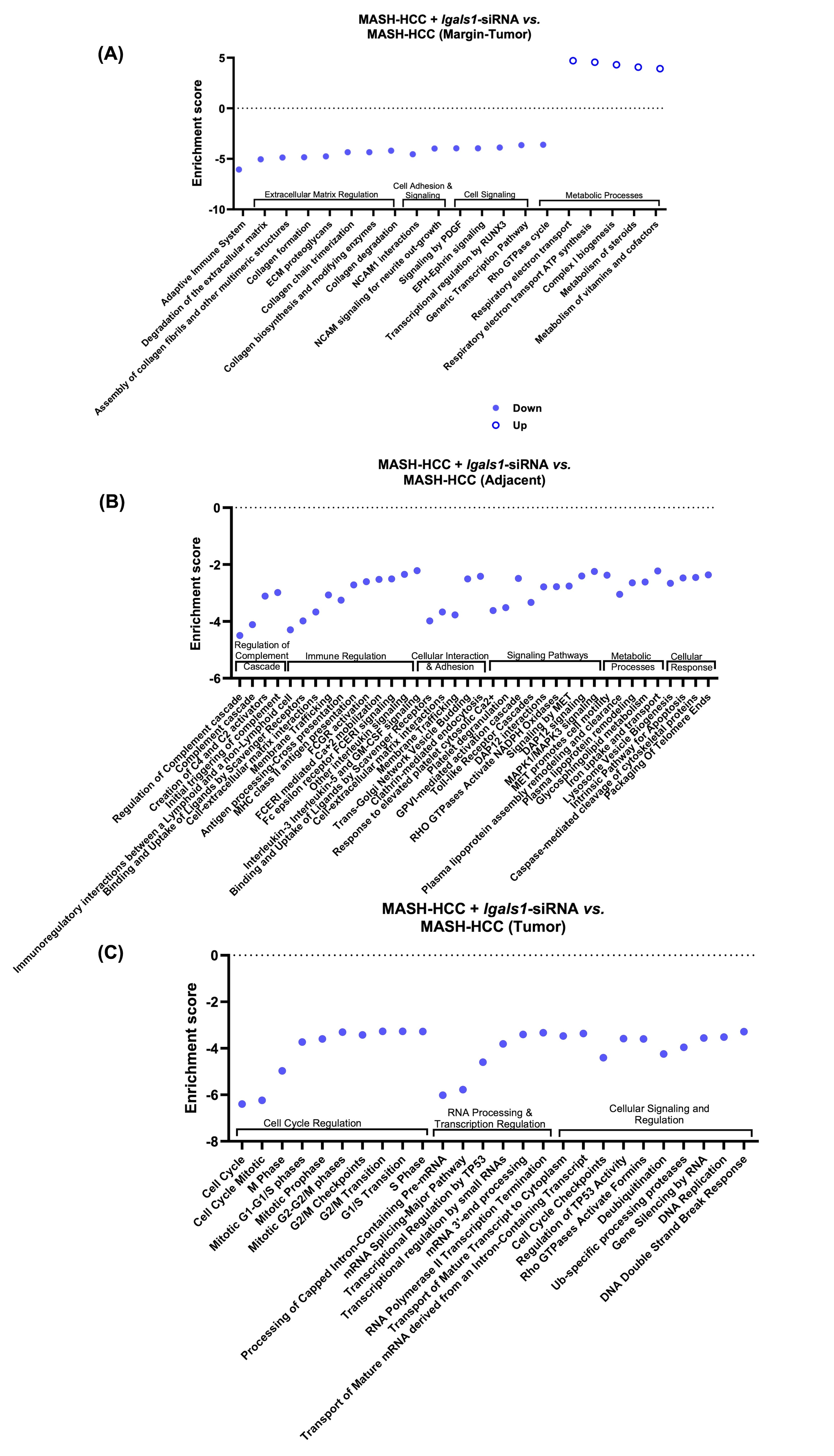

Supplement: Supplementary file 3 — Supplementary Material 3: Supplemental Fig. 2. Pathways that are regulated by Gal-1 silencing in MASH-HCC. (A) tumor margin, (B) adjacent tissues, and (C) inside the tumor. Based on Reactome [file 40364_2024_660_MOESM3_ESM.jpg]
